# Hepcidin: A missing link at the interface of malaria and hypertension

**DOI:** 10.1016/j.ijregi.2024.100463

**Published:** 2024-09-24

**Authors:** Hari Shankar, Auley De, Anat Florentin

**Affiliations:** 1 The Kuvin Center for the Study of Infectious and Tropical Diseases, & Department of Microbiology and Molecular Genetics, Faculty of Medicine, The Hebrew University of Jerusalem, Jerusalem, Israel; 2 Indian Council of Medical Research, New Delhi, India; 3 ICMR-National Institute of Malaria Research, New Delhi, India; 4 Surakhsa Diagnostics Limited, West Bengal, India

**Keywords:** Malaria, Plasmodium, Hepcidin, Hypertension

## Abstract

Malaria and hypertension are seemingly unrelated communicable and non-communicable diseases, both highly pervasive in the global south. There is a debate about the potential connection between these two disorders beyond any obvious factors. The emerging evidence suggests an emerging genetic selection pressure for hypertension in malaria-endemic regions. Furthermore, the impact of the liver hormone hepcidin, which has been overlooked, is significant in the development of hypertension due to malaria. Malaria exposure leads to changes in iron metabolism through the regulation of hepcidin, resulting in iron deficiency and, consequently, the development of hypertension.

## Highlights

• Malaria-associated inflammation overexpresses hepcidin that results in iron deficiency.
• Chronic iron deficiency impacts vascular homeostasis, one of the causes of hypertension.
• Arterial stiffness and high blood pressure have strong association with hepcidin.
• Overexpressed hepcidin potentially aggravate atherosclerosis and hypertension.

## Graphical abstract

**Figure:**
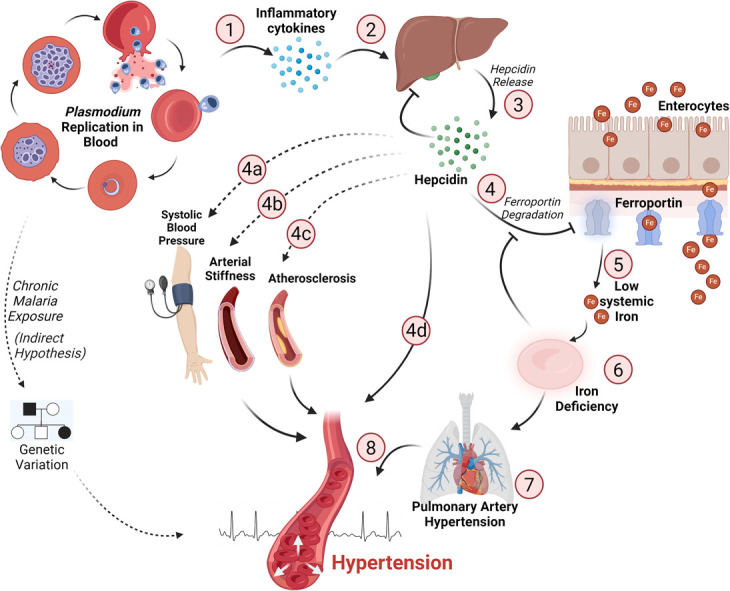

## The global burden of malaria

The year 2022 recorded 249 million global malaria cases and 0.6 million malaria-associated deaths, having a major burden (∼94%) from the World Health Organization (WHO) Africa Region [1]. Malaria is a disease caused by apicomplexan parasites from the genus *Plasmodium*. The disease is spread through infected *Anopheles* mosquitos. The life cycle of the parasite is complex and involves different developmental stages within the mosquito vector, culminating in the infectious sporozoites stage, which reside within the mosquito's salivary glands. During the next blood meal, the sporozoites are released into the human host, infect liver hepatocytes, and, thereafter, move on to invade red blood cells (RBCs). Parasites replication within the RBCs is the source of all clinical manifestation of the disease, including fever and chills [2]. In addition, the continuous rupture of RBCs results in anemia and iron deficiency (ID). However, it is important to note that ID can also be caused by various other factors, such as poor dietary iron absorption, hookworm, or other infections such as HIV, apart from hemolysis [3].

## Hypertension in the global South

Hypertension is a condition described by the persistent rise in blood pressure in the blood vessels. WHO 2023 [1] report on hypertension suggests that around 1.28 billion individuals who are between 30 to 79 years of age are likely to have hypertension, and majority of them (∼66%) of them are residing in low- and middle-income countries. The reported prevalence of hypertension varied across the countries and regions, with the WHO Region of the Americas reported the lowest prevalence of 18%, whereas the WHO African Region experienced 27% of hypertension cases [4]. India has reported 315 million cases of hypertension, accounting for one-third of its overall population [5]. Hypertension is a multifactorial condition, and contributing factors to its consistent rise in Sub-Saharan Africa and India include malnutrition, poor dietary habits, chronic exposure to various infectious diseases in tropical environments, and limited access to health facilities [6]. Several studies have reported a link between hypertension, anemia, and chronic exposure to infectious diseases [7,8]. In these regions, malaria is frequently observed among pregnant women and infants, which increases the risk of gestational hypertension, preeclampsia/eclampsia in the mother, low birth weight, stunting, and increased systolic blood pressure in the early years of life in infants [9]. This is likely to predispose the young ones to develop hypertension at an early age.

## The interplay between malaria and hypertension

The renin-angiotensin system (RAS) is a central hormonal route that regulates blood pressure and hemodynamic stability of circulatory fluid volume. Angiotensin II (Ang II) is the key component of RAS that is responsible for elevated systemic blood pressure. The level of Ang II in the circulation is maintained by two enzymes: angiotensin-converting enzyme (ACE) and ACE-2. Ang II exerts its action by binding with two receptors, i.e. angiotensin type 1 and angiotensin type 2, found on various tissues, including blood vessels, heart, kidneys, and brain. Other than AngII, bradykinin and sphingosine 1-phosphate are crucial biomolecules in blood pressure regulation. Sphingosine 1-phosphate promotes vascular homeostasis by inducing vasoconstriction in vascular smooth muscle cells and vasodilation in vascular endothelial cells. In addition, bradykinin serves as an important vasodilator present in the RAS signaling pathway.

Various epidemiological, functional, genetic, mendelian randomization, and observational studies have raised the global concern on increasing burden and mortality related to hypertension. In addition, it was recently suggested that chronic exposure to malaria could result in hypertension [10]. A possible interplay between malaria and hypertension was suggested by two different hypotheses. The direct hypothesis posits that factors, such as malaria during pregnancy, stunting, malnutrition, and increase in the angiogenic growth factor Ang II, directly contribute to hypertension in malaria-endemic populations [11].

The “indirect” hypothesis suggests that elevated blood pressure in populations living under chronic exposure to malaria may be due to specific genetic variations in the human RAS, such as insertion/deletion in ACE and cytosine/thymine substitution polymorphism [12]. These genetic variations were believed to be the result of positive selection pressure-driven evolutionary adaption and provide protection against severe malaria by elevated Ang II in malaria-endemic regions [13], similar to certain hemoglobinopathies. These two different viewpoints highlight the complex relationship between malaria and hypertension and encourage exploring the underlying pathophysiological mechanism that links one communicable and other non-communicable diseases.

## A potential missing link: Hepcidin

**Figure 1:**
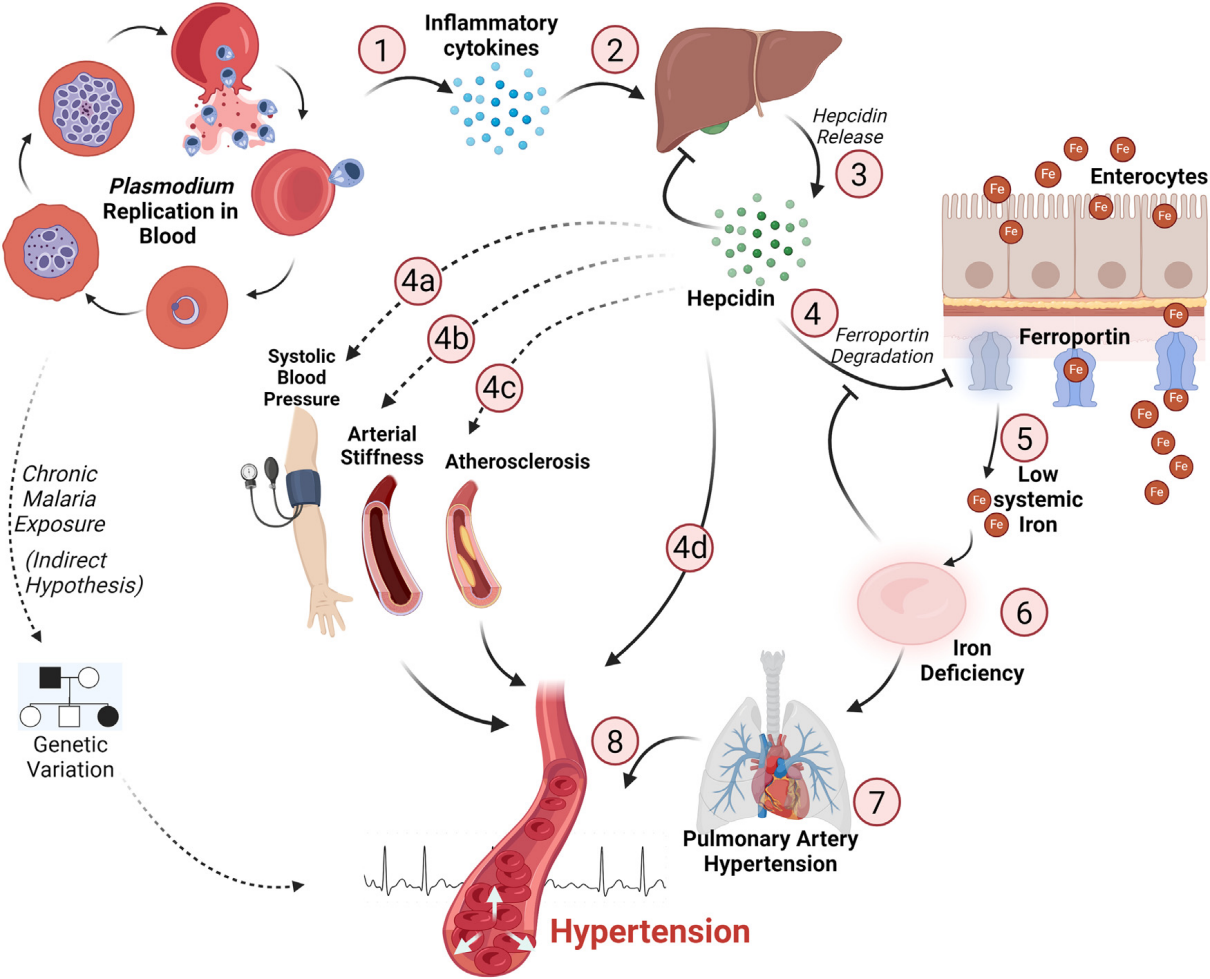
Schematic representation illustrating the role of hepcidin in malaria-induced hypertension. *Plasmodium* infection triggers an inflammatory immune response in the host (1) that results in the release of hepcidin hormone from the liver (2). Hepcidin release (3) causes ferroportin degradation in enterocytes (4) and other cells (not shown here), thereby reducing the systemic iron pool (5), which leads to iron deficiency (6). Hepcidin-mediated iron deficiency directly affects pulmonary artery hypertension (7), ultimately causing hypertension (8) and heart failure. In addition, hepcidin is associated with systolic blood pressure (4a) and is a strong predictor of arterial stiffness (4b), exacerbating atherosclerosis (4c) and hypertension (4d), which are the precursor events to hypertension. The indirect hypothesis refers to genetic variations in human renin-angiotensin genes, which may provide protection against severe malaria. The dashed arrows represent sporadic observations and nonproven hypotheses. The figure was prepared using BioRender.

Asexual replication of the parasite within RBCs triggers the inflammatory cytokine release as an initial defense mechanism. This inflammatory response leads to an augmented expression of hepcidin, a 25-amino-acid peptide hormone released from hepatocytes that is crucial for iron homeostasis in humans (Figure 1). Increased hepcidin expression confines iron inside intestinal cells by breaking down the iron export protein ferroprotein, which is required for transporting dietary iron out of those cells and into the bloodstream. Hepcidin also blocks the absorption of duodenal iron from the diet by downregulating the expression of divalent metal transporter-1. Together, these mechanisms limit the systemic iron pool, ultimately resulting in ID [14] (Figure 1). Conversely, ID and erythropoiesis suppress hepcidin release through a negative feedback loop. Malaria and ID are interrelated major public health issues, commonly observed in the global south. Although ID was proposed to provide some protection against malaria [15], malaria can be one of its causes [3].

Hepcidin seems to be a key player in the interplay between malaria, ID, and the increasing burden of hypertension. The malaria-associated rise in inflammatory cytokines results in the overexpression of hepcidin [16], which may exacerbate atherosclerosis [17] and hypertension [18]. In addition, elevated hepcidin is a strong predictor of arterial stiffness [19] and was found to be associated with systolic blood pressure [20], both of which are the events that precede the development of hypertension (Figure 1).

Several studies have investigated potential links between iron metabolism and the RAS. Some researchers suggest that variations in iron metabolism, such as changes in hepcidin levels, could impact components of the RAS, potentially through pathways involving oxidative stress and inflammation [21]. In addition, there is evidence indicating that ACE inhibitors might influence iron metabolism by potentially reducing hepcidin expression, although the exact mechanisms are not yet fully understood. Lowered hepcidin levels could exacerbate iron metabolism issues in populations with cardiovascular disease, particularly, those residing in malaria-endemic areas and taking ACE inhibitors medication [22]. Chronic ID results in ID anemia (IDA), the persistence of which within the population could potentially contribute to the development of pulmonary arterial hypertension by disrupting pulmonary vascular homeostasis. ID has a direct effect on pulmonary arterial hypertension [23], which eventually leads to heart failure.

## Viewpoint

Malaria-associated inflammation leads to an increased expression of hepcidin and increases systolic blood pressure, arterial stiffness, and ID/IDA in population residing in endemic regions. Chronic ID/IDA has an impact on vascular homeostasis, leading to the onset of hypertension. In contrast, patients with hypertension from an endemic region treated with ACE inhibitors may replenish iron pools due to the downregulatory effect of ACE inhibitors on hepcidin expression. This may render them prone to malaria infection [24].

The elevated levels of hepcidin during malaria infection and its connection to hypertension could help us better understand the interplay of malaria, ID, and hypertension in regions where malaria is common. Possibly, this might shed light on the troubling coexistence of these diseases and inform on rational and effective therapeutic interventions.

## Declarations of competing interest

The authors have no competing interests to declare.
